# Multi-omic analysis of the tumor microenvironment shows clinical correlations in Ph1 study of atezolizumab +/- SoC in MM

**DOI:** 10.3389/fimmu.2023.1085893

**Published:** 2023-07-25

**Authors:** Sandy Wong, Habib Hamidi, Luciano J. Costa, Selma Bekri, Natalia Neparidze, Ravi Vij, Tina G. Nielsen, Aparna Raval, Rajan Sareen, Elisabeth Wassner-Fritsch, Hearn J. Cho

**Affiliations:** ^1^ University of California San Francisco (UCSF) Helen Diller Family Comprehensive Cancer Center, University of California, San Francisco, CA, United States; ^2^ Genentech Inc., South San Francisco, CA, United States; ^3^ O’Neal Comprehensive Cancer Center, The University of Alabama at Birmingham, Birmingham, AL, United States; ^4^ Tisch Cancer Institute, Icahn School of Medicine at Mt. Sinai, New York, NY, United States; ^5^ Yale School of Medicine, New Haven, CT, United States; ^6^ Division of Oncology, Washington University, St. Louis, MO, United States; ^7^ F. Hoffmann-La Roche Ltd., Basel, Switzerland; ^8^ The Multiple Myeloma Research Foundation (MMRF), Norwalk, CT, United States

**Keywords:** atezolizumab, daratumumab, multiple myeloma, biomarkers, tumor microenvironment, multi-omics

## Abstract

Multiple myeloma (MM) remains incurable, and treatment of relapsed/refractory (R/R) disease is challenging. There is an unmet need for more targeted therapies in this setting; deep cellular and molecular phenotyping of the tumor and microenvironment in MM could help guide such therapies. This phase 1b study (NCT02431208) evaluated the safety and efficacy of the anti-programmed death-ligand 1 monoclonal antibody atezolizumab (Atezo) alone or in combination with the standard of care (SoC) treatments lenalidomide (Len) or pomalidomide (Pom) and/or daratumumab (Dara) in patients with R/R MM. Study endpoints included incidence of adverse events (AEs) and overall response rate (ORR). A novel unsupervised integrative multi-omic analysis was performed using RNA sequencing, mass cytometry immunophenotyping, and proteomic profiling of baseline and on-treatment bone marrow samples from patients receiving Atezo monotherapy or Atezo+Dara. A similarity network fusion (SNF) algorithm was applied to preprocessed data. Eighty-five patients were enrolled. Treatment-emergent deaths occurred in 2 patients; both deaths were considered unrelated to study treatment. ORRs ranged from 11.1% (Atezo+Len cohorts, n=18) to 83.3% (Atezo+Dara+Pom cohort, n=6). High-dimensional multi-omic profiling of the tumor microenvironment and integrative SNF analysis revealed novel correlations between cellular and molecular features of the tumor and immune microenvironment, patient selection criteria, and clinical outcome. Atezo monotherapy and SoC combinations were safe in this patient population and demonstrated some evidence of clinical efficacy. Integrative analysis of high dimensional genomics and immune data identified novel clinical correlations that may inform patient selection criteria and outcome assessment in future immunotherapy studies for myeloma.

## 1 Introduction

Most patients with multiple myeloma (MM), a bone marrow (BM) malignancy, experience relapse after initial therapy (1). Relapsed/refractory (R/R) MM remains a challenge (2), and there is a high unmet medical need for more targeted therapies in this setting. Response to MM therapy can vary markedly even among patients at the same disease stage and with comparable clinical characteristics, and tumor intrinsic features such as chromosomal abnormalities, mutations, and gene expression patterns are well-established risk factors (3). An immunosuppressive tumor microenvironment (TME) is essential for MM disease establishment and progression and is implicated in protection of MM cells against anti-tumor therapies (4). Features of the TME, such as CD8+ cells, tumor cells and osteoclasts, may have a considerable impact on the immunologic and clinical efficacy of therapy in MM (5–7). Integration of transcriptomic and proteomic data from both the tumor and the TME can help elucidate the underlying features of this molecular heterogeneity, refining our understanding of the tumor biology (8). Improved understanding of the TME and its impact on therapeutic efficacy may support selection of targeted therapies for specific patient subgroups.

Programmed death-1 (PD-1) is an inhibitory receptor expressed by regulatory T cells (Treg), B cells and some myeloid cell populations (9), and is a validated target for cancer immunotherapy. However, two recent phase 3 studies of PD-1 inhibition via the monoclonal antibody pembrolizumab in patients with treatment-naïve and R/R MM were halted early due to an unfavorable benefit-risk profile (10, 11). Programmed death-ligand 1 (PD-L1), a PD-1 ligand, is expressed on MM cells, with higher levels of expression observed after relapse and with advanced disease (12). PD-L1–expressing immune cells in the TME have been shown to promote MM cell survival and potential immune escape (13). PD-L1 is also expressed on dendritic cells and myeloid-derived suppressor cells in MM patients (14–16); therefore, the anti-PD-L1 monoclonal antibody atezolizumab (Atezo) may have therapeutic application in MM (17). Unlike PD-1 inhibition, PD-L1 inhibition with Atezo may exert a different biologic influence on the TME with more favorable efficacy and tolerability.6 However, PD-L1 inhibition alone is insufficient to induce a durable clinical response (18).

Daratumumab (Dara) is an anti-CD38 monoclonal antibody with multiple modes of immune activity that is approved as a single agent and in combination with proteasome inhibitors (PIs) and immunomodulatory drugs (IMiDs) for patients with new and R/R MM (19, 20). Lenalidomide (Len) and pomalidomide (Pom) are IMiDs that induce myeloma cell death and promote immunity through B- and T-cell activation, dendritic cell maturation and other activities (21, 22). Combinations of these agents with Atezo may be advantageous due to their immunomodulatory activities.

**Figure 1:**
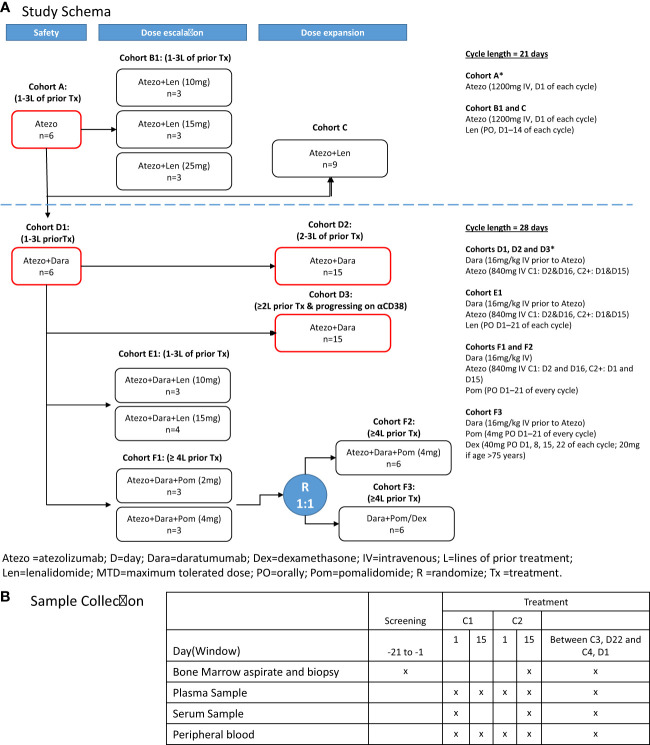
GO29695 **(A)** study design and **(B)** sample collection. *Cohorts included in the correlative analyses. Atezo, atezolizumab; ASCT, autologous stem cell transplant; C, cycle; D, day; Dara, daratumumab; Dex, dexamethasone; IV, intravenously; Len, lenalidomide; PO, orally; Pom, pomalidomide; R, randomization. "x" means the sample was collected at that time point.

We present data from GO29695 (NCT02431208), an open-label phase 1b study to evaluate the safety and efficacy of Atezo alone or in combination with Len, Dara, Dara and Len, or Dara and Pom in patients with MM who are R/R or post-autologous stem cell transplantation (ASCT) with residual disease (**Figure 1**). Exploratory biomarker analyses were performed in patients receiving Atezo monotherapy or Atezo+Dara to validate the pharmacodynamics and mode of action of Atezo (23) and to assess whether Atezo+Dara is additive/synergistic for CD8+ T-cell activation. To gain biological and mechanistic insight into R/R MM in patients receiving Atezo monotherapy or Atezo+Dara, high-dimensional phenotyping was performed and the resultant multiscale data were analyzed integratively using a novel, unbiased, unsupervised machine-learning algorithm based on a patient similarity network (24). These results were compared with clinical data such as patient selection criteria and outcome to investigate correlations between tumor, immune and clinical features.

## 2 Materials and methods

### 2.1 Patients

Patients were enrolled into the following treatment cohorts (**Figure 1A**): Cohort A, Atezo monotherapy; Cohort B1, Atezo+Len (dose escalation); Cohort C, Atezo+Len (post-ASCT patients); Cohort D1, Atezo+Dara (run-in); Cohort D2, Atezo+Dara (expansion); Cohort D3, Atezo+Dara (patients with Dara-refractory disease); Cohort E1, Atezo+Dara+Len (dose escalation); Cohort F1, Atezo+Dara+Pom (dose escalation); Cohort F2, Atezo+Dara+Pom (expansion); and Cohort F3, Dara+Pom+Dex (control).

Key inclusion criteria included previous diagnosis of MM with objective evidence of measurable disease and an Eastern Cooperative Oncology Group performance status (ECOG PS) of ≤2 (**Supplemental Material**; **Supplemental Table 1**).

The study was conducted in accordance with the International Conference on Harmonization guidelines for Good Clinical Practice, and the protocol was approved by the ethics committees of all participating centers and registered at ClinicalTrials.gov (NCT02431208). All patients provided written informed consent.

### 2.2 Treatment

Treatment schedules for each cohort are provided in the **Supplemental Material**; **Figure 1A**. All treatments were given in 21- or 28-day cycles, and continued until loss of clinical benefit, withdrawal, or study end.

### 2.3 Study management

In July 2017, an interim analysis of 2 clinical trials in patients with treatment-naïve or R/R MM, KEYNOTE-18310 and KEYNOTE-185,11 demonstrated inferior overall survival (OS) of pembrolizumab plus Pom/Len vs the control arm (Dex plus Pom/Len), resulting in all studies evaluating anti-PD-(L)1 agents in combination with IMiDs (including GO29695) being placed on partial clinical hold in an FDA class action, despite no new safety signals being observed in this study. In November 2018, with no transformative benefits having been observed, a decision to terminate recruitment to GO29695 was made (patients on study with clinical benefit continued on treatment).

### 2.4 Biomarker assessments

For biomarker and correlative studies, peripheral blood (PB), BM aspirates (BMA) and BM core biopsies (BMBx) were obtained at screening, on treatment at cycle (C) 2 day (D) 15 or prior to C4, and at end of study from patients receiving Atezo monotherapy (Cohort A) or Atezo+Dara (Cohorts D1, D2 and D3) (**Figure 1B**). CD138 positive and negative fractions were separated using EasySep™ Human Whole Blood and BM CD138 Positive Selection Kit II (StemCell Technologies Catalog No. 17887) and StemCell RoboSep 20000 following manufacturer’s instructions.

Flow cytometry was performed using longitudinal PB and BMA to characterize CD8+ cytotoxic T cells using 8 color flow panels at the central laboratory (Covance, NC, USA) according to the laboratory protocol. CD8+ T-cell activation and proliferation were analyzed using %CD8+HLA-DR+Ki-67+.23

Formalin-fixed and decalcified paraffin-embedded tissue sections (4 mm thickness) taken at baseline were used for dual-plex immunohistochemistry (IHC) assay (CD138/tartrate-resistant acid phosphatase [TRAP]) at Histogenics Corp (MA, USA). Osteoclasts were enumerated based on TRAP positivity and morphology (7). Clones used for staining were B-A38-VENTANA for CD138 and EPR15556 ABCAM for TRAP. Briefly, dual-plex immunohistochemistry (IHC) (CD138/CD8, CD8/Ki-67, CD138/osteoclast) were performed using longitudinal CD138+ fraction and bone biopsies, respectively. For IHC, CD138+ cell masses of >5000μm2 were defined as tumor clusters.

RNA sequencing (RNAseq) was performed using longitudinal CD138+ and CD138 (–) enriched BM fractions. RNA isolation, library preparation using the Illumina (CA, USA) TruSeq RNA Access Kit and sequencing using the Illumina HiSeq System (2X50 bp paired-end and target (7)M reads average across samples) were performed at Q Squared Expression Analysis LLC (NC, USA).

Mass cytometry immunophenotyping (39 marker CyTOF panel; Fluidigm, CA, USA) and proteomic profiling (multiplex immuno-oncology assay [Olink]; Olink Proteomics, Uppsala, Sweden) were performed on BM mononuclear cells (BMMC) and BM plasma (**Supplemental Material**; **Supplemental Table 2**). For the CyTOF data, features that had more than 25% of missing values across patients were filtered out. For the remaining missing data, K nearest neighbor imputation was used. For gene expression data, median absolute deviation analysis was performed, and genes with the greatest variability across patients were selected.

For the unbiased integrative analysis, a similarity network fusion (SNF) algorithm (**Supplemental Figure 1**) was applied to pre-processed CyTOF, Olink and RNAseq data, whereby patient similarity networks were constructed for each of the data types using pairwise correlation (SNFTool R package; cran.r-project.org/web/packages/SNFtool) (24). Each patient similarity network was iteratively integrated, making them more similar with each step, using the SNF novel fusion algorithm (with default settings) to generate the integrated network.

Gene set enrichment analysis was implemented to further understand the underlying biological difference between clusters using the gene expression data. One vs all comparison was performed using the hallmark gene set (MSigDB v5.2) (25).

### 2.5 Endpoints

Safety endpoints included incidence, nature, and severity of adverse events (AEs) and serious AEs (SAEs), and occurrence of dose-limiting toxicities. Efficacy endpoints included overall response rate (ORR), duration of response (DOR), progression-free survival (PFS) and OS. Response was assessed by investigators using International Myeloma Working Group 2016 criteria (26).

### 2.6 Data sharing statement

For up to date details on Roche’s Global Policy on the Sharing of Clinical Information and how to request access to related clinical study documents, see here: https://go.roche.com/data_sharing. Requests for the exploratory biomarker data underlying this publication requires a detailed, hypothesis-driven statistical analysis plan that is collaboratively developed by the requestor and company subject matter experts. Direct such requests to basel.nct02431208_biomarker_dr@roche.com for consideration. Anonymized records for individual patients across more than one data source external to Roche cannot, and should not, be linked due to a potential increase in risk of patient re-identification.

## 3 Results

### 3.1 Patient disposition

A total of 85 patients were enrolled (**Figure 1A**) at 16 sites across the US. The study design was re-assessed during the FDA-mandated hold, and Cohorts B, C, and E were voluntarily closed to further recruitment in the amended protocol. Recruitment to Cohorts D and F resumed with the addition of a control arm (Cohort F3) after the hold was lifted. In January 2021, 8 subjects remaining on treatment with clinical benefit were offered alternative drug access, and the study was closed (termination by sponsor with n=36 [42.3%] in survival follow-up; n=34 [40.0%] deaths; n=1 [1.2%] lost to follow-up; n=6 [7.1%]) withdrawals of consent.

### 3.2 Patient demographics

Median age of patients ranged from 56.0 years (range: 50–68) in Cohort F1 to 73.5 years (range: 57–86) in Cohort A (**Table 1**). The majority of patients (71.8%) were male. A third of patients (32.3%) had high-risk cytogenetics (defined as at least one of the following found at screening by local fluorescent *in situ* hybridization (FISH) testing: t (4;14), t(14;16), t(14;20), del17p, 1q21 amplification) and 48.2% had an ECOG PS of 0. In patients with Dara-refractory disease (Cohort D3), the median time from last prior application of Dara to the current study start date was 44.5 days (range: 8–144).

**Table 1:** Patient characteristics.

N (%) unless otherwise stated	Cohort*									
	A (n=6)	B1 (n=9)	C (n=9)	D1 (n=6)	D2 (n=15)	D3 (n=15)	E1 (n=7)	F1 (n=6)	F2 (n=6)	F3 (n=6)
**Median age, years (range)**	73.5 (57–86)	64.0 (56–69)	60.0 (51–67)	58.5 (30–75)	64.0 (52–80)	63.0 (52–86)	63.0 (50–70)	56.0 (50–68)	69.5 (54–76)	62.5 (45–82)
**>65 years of age**	4 (66.7)	3 (33.3)	1 (11.1)	2 (33.3)	5 (33.3)	6 (40.0)	2 (28.6)	1 (16.7)	4 (66.7)	2 (33.3)
**Male**	3 (50.0)	8 (88.9)	5 (55.6)	4 (66.7)	10 (66.7)	13 (86.7)	4 (57.1)	4 (66.7)	5 (83.3)	5 (83.3)
**ECOG PS of 0**	5 (83.3)	5 (55.6)	6 (66.7)	5 (83.3)	4 (26.7)	5 (33.3)	3 (42.9)	3 (50.0)	4 (66.7)	1 (16.7)
**High-risk cytogenetics^†^**	4 (66.6)	1 (11.1)	1 (11.1)	0 (0)	8 (53.3)	5 (33.3)	5 (71.4)	2 (33.3)	1 (16.7)	1 (16.7)
**Refractory to an IMiD and** **a PI**	2 (33.3)	3 (33.3)	0	4 (66.7)	11 (73.3)	14 (93.3)	3 (42.9)	4 (66.7)	3 (50.0)	2 (33.3)
**Median number of prior lines of therapy**	2	3	1	2	2	4	2	4	5	4
**Prior ASCT**	1 (16.7)	7 (77.8)	9 (100)	5 (83.3)	8 (53.3)	5 (33.3)	6 (85.7)	3 (50.0)	1 (16.7)	1 (16.7)

ASCT, autologous stem cell transplant; Atezo, atezolizumab; Dara, daratumumab; Dex, dexamethasone; ECOG PS, Eastern Cooperative Oncology Group Performance Status; IMiD, immunomodulatory drug; Len, lenalidomide; PI, proteasome inhibitor; Pom, pomalidomide.

*Cohort A: Atezo monotherapy; B1: Atezo+Len; C: Atezo+Len; D1/D2/D3: Atezo+Dara; E1: Atezo+Dara+Len; F1/F2: Atezo+Dara+Pom; F3: Dara+Pom+Dex

^†^High-risk cytogenetics defined as at least one of the following found at screening: t(14;16), t(14;20), del17p, chromosome 1 amplification.

### 3.3 Safety

All patients in all cohorts experienced at least one AE (**Table 2**). The most common AEs (>25%) in the safety-evaluable population (n=79) were fatigue (30/79 patients [38.0%]), diarrhea (27/79 [34.2%]), neutropenia (26/79 [32.9%]), thrombocytopenia (n=24/79 [30.4%]), and anemia (22/79 [27.8%]) (**Supplemental Table 3**). Grade 3–5 AEs were reported in 47 patients (59.5%); the most common (>10%) were neutropenia (15/79 patients [19.0%]) and pneumonia (9/79 [11.4%]). One event of auto-immune hepatitis was reported. Other potential immune-related events have been reported such as hypothyroidism (4 events), and secondary adrenocortical insufficiency and pancreatitis (one event each; no confirmation of the underlying cause of these events was collected). Two patients (both in Cohort D2) experienced a Grade 5 (fatal) AE (multiple organ dysfunction syndrome and intracranial hemorrhage). Both events were considered by the investigator to be due to disease progression and unrelated to Atezo or Dara. No new treatment-emergent SAEs were identified. Details of AEs that led to withdrawal from treatment in each cohort are provided in the **Supplemental Material**.

**Table 2:** Safety summary.

N (%)	Cohort*										
	A (n=6)	B1 (n=9)	C (n=9)	D1 (n=6)	D2 (n=15)	D3 (n=15)	E1 (n=7)	F1 (n=6)	F2 (n=6)	F3 (n=6)	All patients (n=85)
**At least one AE, any grade**	6 (100)	9 (100)	9 (100)	6 (100)	15 (100)	15 (100)	7 (100)	6 (100)	6 (100)	6 (100)	85 (100)
**At least one SAE**	1 (6.7)	2 (22.2)	1 (11.1)	2 (33.3)	6 (39.9)	2 (13.4)	3 (42.9)	4 (66.7)	4 (66.7)	5 (83.3)	30 (35.3)
**Grade 3–5 AEs**	1 (16.7)	6 (66.7)	2 (22.2)	4 (66.7)	9 (60.0)	6 (40.0)	6 (85.7)	6 (100)	6 (100)	6 (100)	52 (61.2)
**Grade 5 AEs**	0	0	0	0	2 (13.3)	0	0	0	0	0	2 (2.4)
**AE leading to withdrawal from Atezo**	0	0	0	0	2 (13.3)	1 (6.7)	1 (14.3)	0	1 (16.7)	0	5 (5.9)
**AE leading to withdrawal from Len**	0	0	0	0	0	0	1 (14.3)	–	–	–	1 (1.2)
**AE leading to withdrawal from Dara**	–	–	–	0	2 (13.3)	1 (6.7)	1 (14.3)	0	1 (16.7)	1 (16.7)	6 (7.1)
**AE leading to withdrawal from Pom**	–	–	–	–	–	–	–	0	2 (33.3)	1 (16.7)	3 (3.5)
**AE leading to withdrawal from Dex**	–	–	–	–	–	–	–	0	1 (16.7)	1 (16.7)	2 (2.4)

AE, adverse event; Atezo, atezolizumab; Dara, daratumumab; Dex, dexamethasone; Len, lenalidomide; Pom, pomalidomide; SAE, serious adverse event.

*Cohort A: Atezo monotherapy; B1: Atezo+Len; C: Atezo+Len; D1/D2/D3: Atezo+Dara; E1: Atezo+Dara+Len; F1/F2: Atezo+Dara+Pom; F3: Dara+Pom+Dex. "-" means not applicable.

Median duration of exposure to Atezo was between 1.4 months (range: 0.0–12.4) in Cohort D3 to 41.5 months (range: 8.8–53.8) in Cohort D1 (**Supplemental Table 4**). The median number of Atezo doses received ranged from 4 in Cohorts C and D3 to 81 in Cohort D1.

### 3.4 Efficacy

Median follow-up ranged from 23.7 months in Cohort F3 to 63.0 months in Cohort A (**Table 3**). The ORR ranged from 83.3% in Cohorts F1 and F3 to 11.1% in Cohorts B1 and C. No responses were observed in Cohorts A or D3 (**Table 3**).

**Table 3:** Summary of response and survival.

N (%) unless otherwise stated	Cohort*									
	A (n=6)	B1 (n=9)	C (n=9)	D1 (n=6)	D2 (n=15)	D3 (n=15)	E1 (n=7)	F1 (n=6)	F2 (n=6)	F3 (n=6)
**Median follow-up, months (95% CI)**	63.0 (NE, NE)	57.0 (50.6, 58.6)	37.9 (19.8, 52.9)	50.0 (50.2, 51.7)	24.2 (22.8, 32.8)	28.4 (26.0, 32.5)	41.4 (41.4, 42.8)	40.4 (32.2, 42.0)	26.5 (23.4, 31.7)	23.7 (22.4, 29.0)
**ORR, %**	0	11.1	11.1	66.7	33.3	0	57.1	83.3	50.0	83.3
**sCR**	0	0	0	2 (33.3)	0	0	1 (14.3)	0	0	1 (16.7)
**CR**	0	0	0	0	0	0	1 (14.3)	1 (16.7)	0	0
**VGPR**	0	1 (11.1)	1 (11.1)	1 (16.7)	2 (13.3)	0	1 (14.3)	3 (50.0)	2 (33.3)	1 (16.7)
**PR**	0	0	0	1 (16.7)	3 (20.0)	0	1 (14.3)	1 (16.7)	1 (16.7)	3 (50.0)
**MR**	0	0	1 (11.1)	1 (16.7)	1 (6.7)	0	2 (28.6)	0	3 (50.0)	0
**SD**	6 (100)	7 (77.8)	5 (55.6)	1 (16.7)	7 (46.7)	8 (53.3)	0	1 (16.7)	0	0
**PD**	0	1 (11.1)	2 (22.2)	0	1 (6.7)	7 (46.7)	0	0	0	1 (16.7)
**Missing or unevaluable**	0	0	0	0	1 (6.7)	0	1 (14.3)	0	0	0
**Median DOR, months (95% CI)**	NE	45.2 (NE, NE)	NE	NE	5.2 (1.9, NE)	NE	NE	NE	NE	16.5 (1.9, NE)
**Median PFS, months (95% CI)**	2.1 (2.1, 3.8)	3.5 (2.4, 3.9)	6.9 (2.7, NE)	28.9 (21.1, NE)	3.7 (2.8, 5.0)	1.2 (0.9, 1.9)	20.6 (10.8, NE)	NE	12.3 (7.4, NE)	20.7 (2.7, NE)
**Median OS, months (95% CI)**	46.0 (38.8, 57.9)	NR	NR	NE	18.5 (13.1, NE)	22.3 (9.6, NE)	21.9 (10.8, NE)	NR	NR	NR

Atezo, atezolizumab; CI, confidence interval; CR, complete response; Dara, daratumumab; Dex, dexamethasone; DOR, duration of response; Len, lenalidomide; MR, minimal response; NE, not evaluable; NR, not reached; ORR, overall response rate; OS, overall survival; PFS, progression-free survival; PD, progressive disease; Pom, pomalidomide; PR, partial response; sCR, stringent complete response; SD, stable disease; VGPR, very good partial response.

*Cohort A: Atezo monotherapy; B1: Atezo+Len; C: Atezo+Len; D1/D2/D3: Atezo+Dara; E1: Atezo+Dara+Len; F1/F2: Atezo+Dara+Pom; F3: Dara+Pom+Dex.

**Figure 2:**
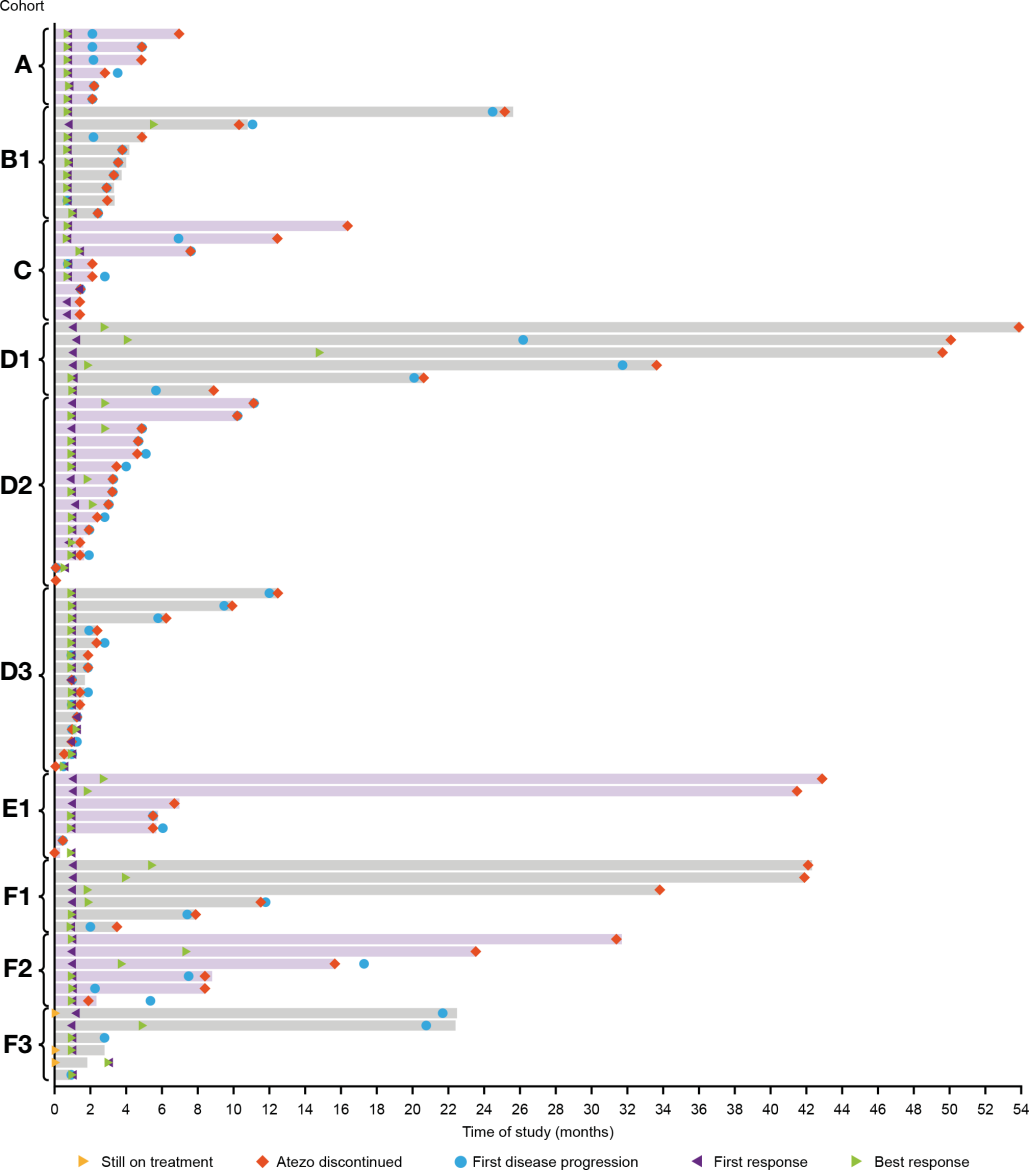
Duration of response in all cohorts. D1/D2/D3: Atezo+Dara; E1: Atezo+Dara+Len; F1/F2: Atezo+Dara+Pom; F3: Dara+Pom+Dex. Atezo, atezolizumab; Dara, daratumumab; Dex, dexamethasone; Len, lenalidomide; Pom, pomalidomide.

Median DOR was 45.2 months, 5.2 months and 16.5 months for Cohorts B1, D2 and F3, respectively and was not estimable for the remaining cohorts (**Figure 2**; **Table 3**). Median PFS ranged from 1.2 months in Cohort D3 to 28.9 months in Cohort D1 (**Table 3**). Median OS ranged from 18.5 months in Cohort D2 to 46.0 months in Cohort A (**Table 3**).

A small number of patients treated with Atezo+Dara, Atezo+Dara+Len, or Atezo+Dara+Pom (Cohorts D1, E1 and F1, respectively) demonstrated deep and durable responses, with 2 patients in Cohort D1 and 1 patient each in Cohorts E1 and F1 achieving long-lasting sCR. No responses to Atezo+Dara were observed in patients with Dara-refractory disease (Cohort D3), and the promising results in Cohort D1 did not propagate in the limited number of patients in D2 (ORR, 33%; CR, 0%) prior to study closure, with the caveats that patients in this cohort were older (median age: 64 years vs 59 years) and had a worse cytogenetic profile (high-risk cytogenetics: 73% vs 67%) than those in D1.

### 3.5 Biomarker and multi-omics assessments

#### 3.5.1 Pharmacodynamic response to Atezo monotherapy

In Cohort A, on-treatment T-cell activation and proliferation were observed in PB at C1D15 vs baseline (**Supplemental Figure 2**) but not in BMAs at C4D1. All Dara-naïve patients (including those receiving Atezo monotherapy), but not those with Dara-refractory disease, showed an on-treatment increase in %CD8+HLA-DR+Ki-67+ cells in PB vs baseline (**Supplemental Figure 3A**). In BMAs, an increase in %CD8+HLA-DR+Ki-67+ cells was observed in Dara-naïve patients with clinical response to Atezo+Dara (sensitive), but not in patients with Dara-resistant or Dara-refractory disease (**Supplemental Figure 3B**).

#### 3.5.2 Osteoclast density

Immunohistochemical analysis of baseline BMBx demonstrated that osteoclast density in the tumor region was higher in non-responders receiving Atezo+Dara vs responders (**Supplemental Figure 4A**). Patients with Dara-refractory disease also demonstrated significantly higher osteoclast density in the tumor region at baseline vs Dara-naïve patients (**Supplemental Figure 4B**).

#### 3.5.3 Integrative clustering analysis

**Figure 3:**
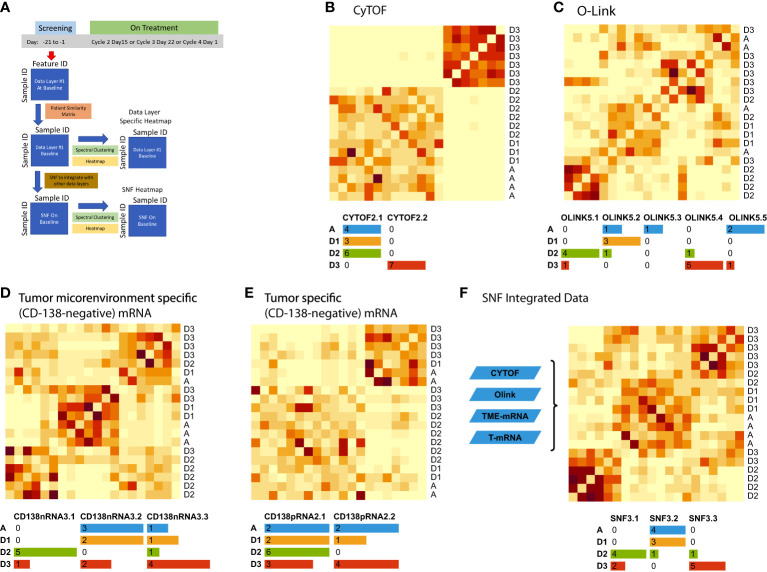
Unsupervised integrative clustering analysis at baseline schema **(A)** using **(B)** CyTOF, **(C)** Olink, **(D)** microenvironment-specific (CD138-negative), **(E)** tumor-specific (CD138-positive), and **(F)** SNF integrated data. Cohort A: Atezo monotherapy; B1: Atezo+Len; C: Atezo+Len; D1/D2/D3: Atezo+Dara; E1: Atezo+Dara+Len; F1/F2: Atezo+Dara+Pom; F3: Dara+Pom+Dex SNF, similarity network fusion; T, tumor; TME, tumor microenvironment. The heatmaps show clustered similarity matrices, with higher values depicted with darker shades indicating greater similarity between patients. In addition, the number of patients per cohort are color coded in the tables beneath each heatmap.

**Figure 4:**
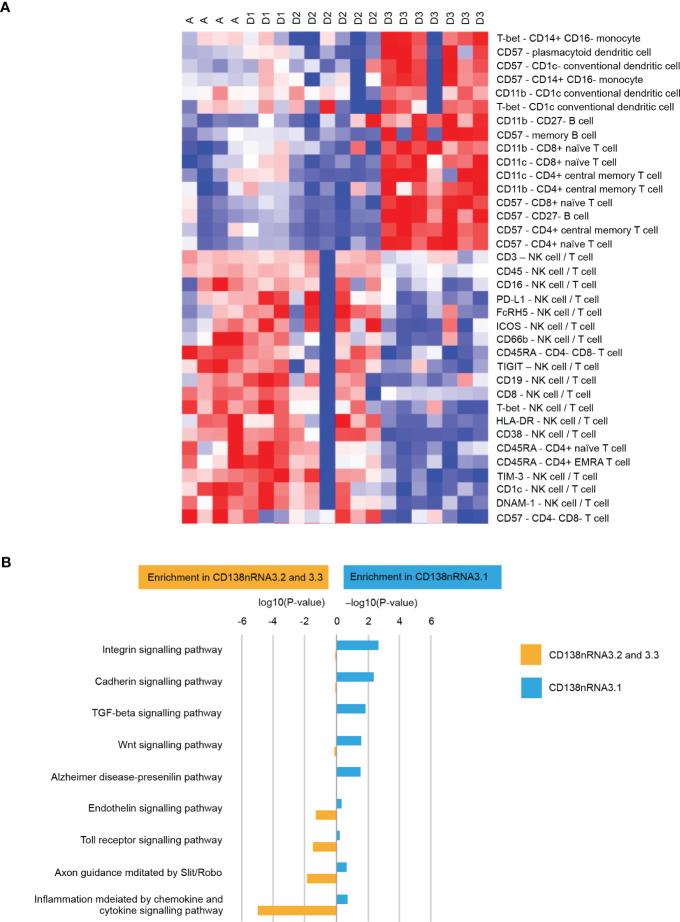
**(A)** Cellular phenotypes associated with cluster D3 (patients with Dara-refractory disease) as identified by CyTOF Pathway Enrichment Analysis and **(B)** Pathway Enrichment Analysis. One vs all comparison was performed for cluster CD138nRNA3.1. Significant genes (t-test p-value<0.01) that had higher expression in CD138nRNA3.1 (enrichment in CD138nRNA3.1) or other clusters (enrichment in CD138nRNA3.2 and 3.3) were subjected to Pathway Enrichment Analysis using the Panther Overrepresentation Test (Pantherdb.org). DNAM-1, DNAX accessory molecule-1; FcRH5, Fc receptor homolog 5; HLA-DR, human leukocyte antigen-DR isotype; ICOS, inducible T-cell co-stimulator; NK, natural killer; PD-L1, programmed death-ligand 1; T-bet, T-box expressed in T cells; TIGIT, T cell immunoreceptor with Ig and ITIM domains; TI<-3, T cell immunoglobulin and mucin-3.

We applied deep molecular and cellular phenotyping (CyTOF, Olink proteomics, BMMC and MM RNA sequencing) to interrogate the TME. SNF was used to integrate these four distinct data types collected at baseline from 20 patients. SNF analyses multiple layers of data to gain a more holistic understanding, capturing shared and complementary information to distinguish true biological signals from noise by weighing down the weak data layer-specific information captured by single-layer analysis. This enables more accurate molecular classification with a significantly lower sample size requirement than single -omics methods (27). Patient similarity networks obtained from CyTOF immunophenotyping of BMMC (analytes in assay, n=897; patient samples analyzed, n=20), Olink proteomic analysis of BM plasma (analytes, n=92; patient samples, n=20), microenvironment-specific gene expression (BMMC CD138-negative mRNA; genes selected for analysis, n=2500) and tumor-specific gene expression (BM CD138+ myeloma cell mRNA; n=2500) features were computed individually to derive individual data layer specific group and then fused together using SNF to derive the clusters of patients from the integration of the four data layers (**Figure 3A**). For the individual data layer, CyTOF analysis clearly distinguished patients with Dara-refractory disease (Cohort D3) from Dara-naïve patients (Cohort D2; **Figure 3B**). Significant enrichment of CD57+ T-cells was observed in the Dara-refractory cluster vs the other clusters (**Figure 4B**). Olink data distinguished Cohort D2 from Cohort D3 (**Figure 3C**), although not as clearly as observed with CyTOF data. TME-specific mRNA gene expression analysis identified three patient groups (**Figure 3D**), whereas tumor-specific mRNA gene expression analysis separated the patients into two (**Figure 3E**), indicating that gene expression from these two compartments describes different layers of the patient. The TME-specific mRNA gene expression data layer enriched for patients in Cohort D2 in the CD138nRNA3.1 cluster. We compared the Cohort D2-enriched cluster (CD138nRNA3.1) with the remaining clusters and observed a significant enrichment of integrin signal pathway genes (p=0.002) in genes that had higher expression in that cohort (**Figure 4B**; **Supplemental Table 5**). The fused SNF-derived network was able to integrate information from all four layers of data types, separating patients into three groups which distinguished Cohort D2 from Cohort D3 (Dara-refractory patients) and Cohorts A and D1 (Dara-naïve patients; **Figure 3F**).

The SNF derived groups were compared with the CyTOF derived group with regards to association with overall survival and duration of response (DOR) (**Supplemental Figure 5**). The SNF derived groups were more predictive of both OS (log-rank p-value=.16, median OS in days SNF3.1 = 583.5, SNF3.2 = 1606 and SNF3.3=NA) and DOR (log-rank p-value=0.27, median DOR in days SNF3.1 = 78, SNF3.2 = 126 and SNF3.3 = 45) than the CyTOF derived group (OS log-rank p-value=0.36, median OS in days CyTOF2.1 = 1606 and CyTOF2.2 = 614; DOR log-rank p-value=0.28, median DOR in days CyTOF2.1 = 102 and CyTOF2.2 = 60).

**Figure 5:**
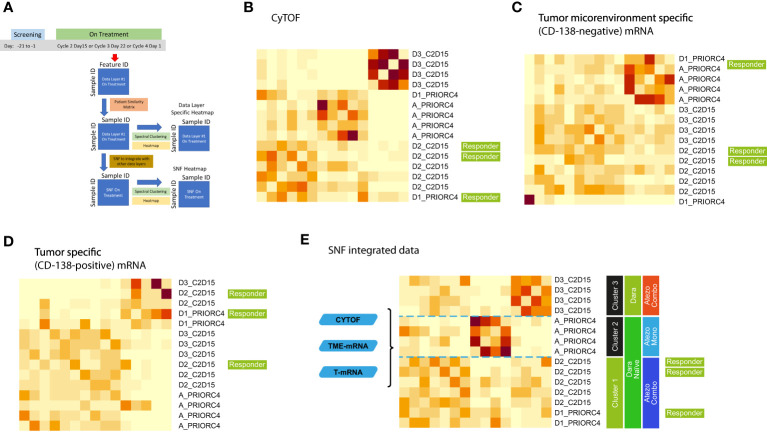
Integrative analysis of on-treatment samples schema **(A)** using **(B)** CyTOF, **(C)** microenvironment-specific (CD138-negative), **(D)** tumor-specific (CD138-positive), and **(E)** SNF data. Cohort A: Atezo monotherapy; B1: Atezo+Len; C: Atezo+Len; D1/D2/D3: Atezo+Dara; E1: Atezo+Dara+Len; F1/F2: Atezo+Dara+Pom; F3: Dara+Pom+Dex. Atezo, atezolizumab; C, cycle; Combo, combination; D, day; Dara, daratumumab; Mono, monotherapy; SNF, similarity network fusion. The heatmaps show clustered similarity matrices, with higher values depicted with darker shades indicating greater similarity between patients.

The unsupervised SNF algorithm was applied to data collected on-treatment from 15 patients, fusing the patient similarity networks obtained from CyTOF, CD138-negative mRNA and CD138-positive mRNA features (**Figure 5A**). On-treatment CyTOF data separated Cohort D3 (Dara-refractory; combination treatment) from the remaining cohorts (**Figure 5B**). The TME-specific mRNA gene expression analysis identified two patient groups (**Figure 5C**) and was able to distinguish Cohort A (Atezo monotherapy). The tumor-specific mRNA gene expression analysis also separated the patients into two groups (**Figure 5D**), but these were not strongly associated with any specific treatment. The fused network integrated information from all three data layers was able to clearly isolate the treatment cohorts (**Figure 5E**). Cluster 1 included patients from Cohorts D1 and D2 (Dara-naïve and receiving Atezo combination therapies), and included the 3 responders. Cluster 2 included Dara-naïve patients receiving Atezo monotherapy (Cohort A). Cluster 3 included patients with Dara-refractory disease receiving Atezo combination therapy (Cohort D3).

#### 3.5.4 Pairwise analysis

Pairwise analysis was performed to assess the dynamic range of informative elements across the entire set of TME cellular and molecular features in each SNF patient group. We compared baseline and on-treatment data from the three assays described above (13 patients). In the CyTOF data layer, patients in cluster 1 exhibited relatively increased expression of NKG2D in naïve, memory CD4 T cells and Tregs, which normally express very low levels of NKG2D. It is possible that their low frequency overstates the high-fold change in the pairwise analysis.

**Figure 6:**
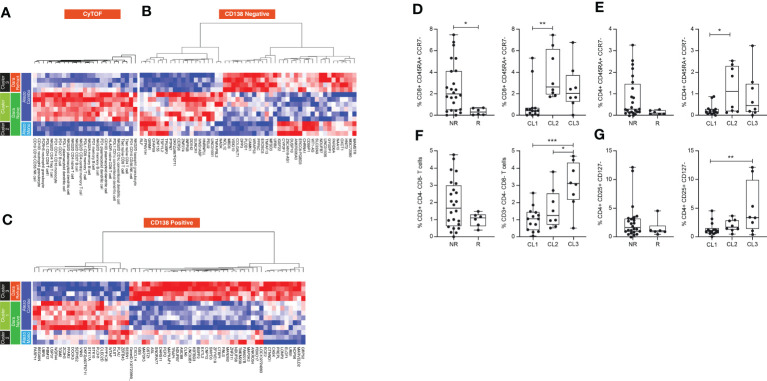
Pairwise longitudinal analysis of **(A)** CyTOF, **(B)** microenvironment-specific (CD138-negative), and **(C)** tumor-specific (CD138-positive) data, and comparison of cell frequency in responders and non-responders of **(D)** CD8 TEMRA cells, **(E)** CD4 TEMRA cells, **(F)** DN T cells and **(G)** Tregs, and their distribution in each cluster. Mann-Whitney and Tukey’s multiple comparisons tests, *P<0.05; **P<0.01; *** P<0.001. Atezo, atezolizumab; CL, cluster; Dara, daratumumab; DN, double-negative; Mono, monotherapy; NK, natural killer; NR, non-responder; PD, programmed death; PDL, programmed death ligand; R, responder; Refract., refractory; TEMRA, terminally differentiated effector memory T cell; Treg, regulatory T cell.

A relative over-representation of T-cell activation markers such as HLADR, T-bet and PD-1, and CD103+ dendritic cells (specialized in cross-priming) was observed in cluster 1 vs the other two clusters, suggesting an activated adaptive immune signature that may be associated with response (**Figure 6A**). Analysis of non-responders vs responders highlighted an increased proportion of terminally-differentiated CD4+ and CD8+ T cells (TEMRA), as well as double-negative T cells and Treg, suggesting an exhausted T-cell phenotype and a suppressive TME in non-responders. These cell types were enriched in cluster 3, the Dara-refractory group (**Figures 6D-G**). RNA sequencing data demonstrated that genes associated with T-cell activation and proliferation such as HNRNPLL, PTPN7 and PEIL2 were upregulated in cluster 1 (Dara-naïve) relative to cluster 3 (Dara-refractory). In contrast, genes upregulated in cluster 3 are associated with regulation of cell growth (PP5C) and the p53 pathway (URB3, WRAP53), and cell adhesion (VSIG10). Although differential expression was observed between the two Dara-naïve clusters, we could not discern a characteristic signature (**Figure 6B**). Similar patterns were observed in the tumor mRNA gene expression analysis (**Figure 6C**). Genes involved in cell growth, survival and resistance to apoptosis (MAPK2,MAPK5) as well as genes associated with cell-cell adhesion (MARVELD2, CTNDD1) were highly upregulated in cluster 3, consistent with tumor resistance and patient outcome in this group.

#### 3.5.5 Gene set enrichment analysis

**Figure 7:**
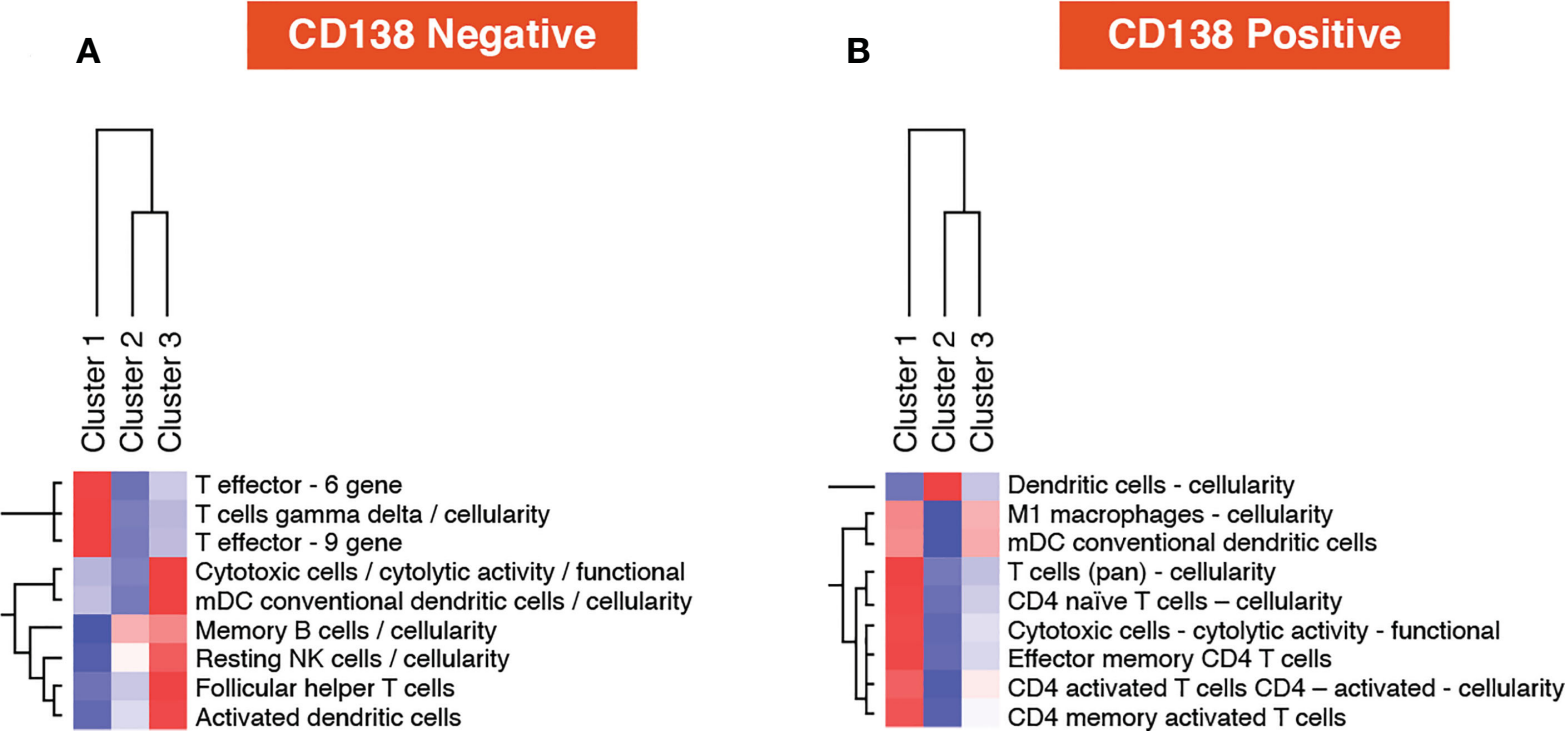
Post-treatment one vs all gene set analysis using **(A)** microenvironment-specific (CD138-negative), and **(B)** tumor-specific (CD138-positive) data. mDC, myeloid dendritic cells; NK, natural killer.

In the TME-specific gene expression (CD138-negative) data layer, cluster 1 (Dara-naïve patients) was enriched for the T cell gamma delta gene signature. The T effector 6 gene signature was also higher in cluster 1 (**Figure 7A**). In the tumor-specific mRNA gene expression (CD138+) data layer, no unique signature was associated with cluster 1. The strongest increase in gene signatures was observed in cluster 2 (Dara-naïve patients receiving Atezo monotherapy), and the dendritic cell gene signature was significantly increased in cluster 3 (Dara-refractory disease; **Figure 7B**).

## 4 Discussion

This study showed that Atezo alone and in combination with Dara and IMiDs was safe in myeloma patients. There was no safety signal and the observed safety profiles of the combinations were consistent with those of the individual agents. The correlative analysis showed that Atezo had the expected immunologic effects in the T cell compartment even in the absence of clinical activity, and multi-omic SNF analysis of cellular and molecular data from the TME recapitulated the cohorts with high fidelity and revealed novel correlations with clinical outcome.

Biomarker data from Cohort A suggest that Atezo promotes T-cell activation as early as C1D15, and all Dara-naïve patients showed an on-treatment increase in T-cell activation and proliferation in blood, although the same effect was not observed in patients with Dara-refractory disease. Evaluation of BMAs and osteoclasts allowed stratification of non-responders and responders, and analysis of baseline osteoclast levels was able to differentiate Dara-naïve patients from those with Dara-refractory disease. This suggests that osteoclasts may contribute to the immunosuppressive TME that inhibits T-cell function as reported previously (28), consistent with the observed lack of T-cell activation observed in Dara-refractory disease.

Patients in the Dara-containing Cohorts (D1, E1 and F1) demonstrated deep and durable responses, however, patients with Dara-refractory disease did not respond to Dara + Atezo (Cohort D3). Similarly, patients in D2 did not recapitulate the promising responses seen in D1, although differing patient selection criteria may have biased this cohort to older patients and greater frequency of high-risk cytogenetics. Furthermore, baseline CyTOF data revealed that patients in the Dara-refractory cluster had higher numbers of the senescent-type CD57+ T cells, which are known to be unable to inhibit growth of malignant cells and may have negatively affected the immune response to tumor antigens (29, 30). The TME-specific mRNA gene expression data layer enriched for patients in Cohort D2 in cluster CD138nRNA3.1 which had significantly higher expression of integrin signal pathway genes, indicating that integrin signaling may represent a mechanism of resistance to Dara+Atezo and a potential therapeutic target. Integrins are expressed on TME cells, such as fibroblasts and endothelial cells, which participate in the cross-talk relevant to tumor progression, specifically functioning as mediators of the interaction between tumor cells, cancer-associated fibroblasts and the matricellular proteins relevant to tumor progression (31). Although integrin-targeting cancer therapies are not effective alone, there is emerging evidence that a combination approach together with anti-PD-1 can improve response. (32) These findings show that the selection criteria reflected not only the clinical status of these patients, but also the state of the TME.

High-dimensional immune profiling and integrative clustering analysis of baseline and on-treatment samples revealed novel associations with outcome in both Dara-naïve and Dara-refractory R/R MM treated with Atezo+Dara. Pairwise analysis comparing matched treatment samples to baseline identified potential biomarkers, such as NKG2D and T cell activation/exhaustion genes, that were differentially expressed across patient subgroups. Similarly, the one-vs-all comparisons of the hallmark gene sets in the CD138–negative gene expression data layer revealed that cluster 1 is enriched for the T cell gamma delta gene signature and the T effector 6 gene signature. This is in line with previous studies, in which these gene signatures have been associated with response to cancer immunotherapy treatment (33, 34). A recent study of gene expression in patients with R/R MM revealed that a number of immune response-regulating genes were the most downregulated pathways in Dara-resistant vs Dara-naïve patients, further supporting the hypothesis that changes in the immune compartment of the TME may play a role in resistance to Dara (35). An integrated multi-omics approach has previously been proposed in patients with R/R MM, in which patients were treated based on their tumor DNA and RNA sequencing data, with results demonstrating feasibility and early efficacy (36). The authors noted that the addition of profiling of the TME (using single-cell RNAseq or mass cytometry) into their platform may further improve the specificity of the approach. SNF analyses in this study confirm that integration of high-dimensional tumor and TME data is feasible and can identify potential biomarkers of resistance or response in the setting of combination immunotherapy.

Given the complexity of the immune response to cancer and the proliferation of immunotherapeutic strategies in MM, it is critical to identify predictive and prognostic biomarkers to guide treatment. High-dimensional single-cell and whole transcriptomic approaches profiling the tumor and the microenvironment as applied in this study are being more readily applied clinically (37). Conventional single cell diagnostics such as FISH and flow cytometry are limited in their ability to capture the diversity of cell types and states. High-dimensional flow cytometry and mass cytometry (CyTOF) have made it possible to detect significantly larger numbers of proteins in thousands of cells, allowing deeper molecular characterization of cell populations in detail in an increasingly cost-effective manner. These assays bring their own set of challenges; for example, gold standard methods of analyzing flow cytometry data are inefficient and unreliable when applied to high-dimensional data. The FlowCAP (Flow Cytometry: Critical Assessment of Population Identification Methods) Consortium has recognized that high-dimensional assays such as CyToF require algorithm-based clustering approaches that have the advantages of being unbiased and automated (38). Biomarkers derived from high dimensional assays and data analyses may eventually inform treatment (39).

## 5 Conclusion

These findings demonstrate that anti-PD-L1 therapy with Atezo (as monotherapy and in combination) is safe and has the expected immunologic activity in MM patients. Integrated SNF analysis of high-dimensional immunophenotyping data demonstrated that patient selection based on clinical criteria had a significant impact on the immunologic state of the TME at baseline. Novel correlations with clinical outcome suggest that T-cell associated factors may predict response to treatment. With the rapid introduction of novel immune therapies in MM including CAR-T cells and bispecific antibodies, addressing mechanisms of immune escape is even more critical. Therefore, we believe that the safety and efficacy data reported here support further investigation of PD-L1 checkpoint inhibition combinations in myeloma. This rational immune-oncology approach may enhance understanding of myeloma-TME biology and promote biomarker-driven treatment.

## Data availability statement

The data presented in the study are deposited in European Genome-Phenome Archive (EGA) repository, accession number EGAS00001007286.

## Ethics statement

The study was conducted in accordance with the International Conference on Harmonization guidelines for Good Clinical Practice, and the protocol was approved by the ethics committees of all participating centers and registered at ClinicalTrials.gov (NCT02431208). All patients provided written informed consent. The patients/participants provided their written informed consent to participate in this study.

## Author contributions

HC, SB, TN, EW-F and HH contributed to the conception and design of the study. SW, LCJ, NN, and RV contributed to the provision of study materials or patients. HH, HC, SB, TN, and EW-F collected and assembled the data. All authors analyzed and interpreted the data, contributed to the writing of the manuscript, and provided final approval of the manuscript.
