# Supplementary material for: Multi-omic analysis of the tumor microenvironment shows clinical correlations in Ph1 study of atezolizumab +/- SoC in MM
Source: Front Immunol. 2023 Jul 25;14:1085893. doi: 10.3389/fimmu.2023.1085893 (PMC10408441; doi:10.3389/fimmu.2023.1085893)

***Supplementary Material***

# Methods

## Inclusion and exclusion criteria

Patients enrolled in Cohorts D1, D2, E1, F1, F2 and F3 must not have received prior treatment with an anti-CD38 therapy. Patients in Cohorts A, B1, C, D1 and E1 must have received 1–3 prior lines of therapy. Patients in Cohort C must have sufficient recovery from first or second ASCT within 60–120 days of transplant. Patients in Cohort D2 must have received 2–3 prior lines of therapy and be refractory to their last line of treatment. Patients in Cohort D3 must have received ≥2 prior lines of treatment, be refractory to a PI and an IMiD, and progressed on Dara in the last line. Patients in Cohorts F1, F2 and F3 must have received ≥4 prior lines of therapy and be refractory to their last line. Key exclusion criteria included prior treatment with chimeric antigen receptor (CAR)-T cell therapy or prior allogeneic stem cell transplant.

## Treatments

Patients in Cohorts A–C received atezolizumab (Atezo) 1200 mg intravenously (IV) on Day (D) 1 of each 21-day cycle. Patients in Cohorts D–F (except those in F3) received Atezo 840 mg IV on Cycle (C) 1 D2 and C1D16, and on D1 and D15 of each 28-day cycle thereafter. Patients in Cohorts D1, D2, D3, E1, F1, F2 and F3 received daratumumab (Dara) 16 mg/kg IV on D1, D8, D15, and D22 of C1 and C2, on D1 and D15 of C3–6, and on D1 of each 28-day cycle thereafter. Patients in Cohort B1 received lenalidomide (Len) 10, 15 or 25 mg orally (PO) on D1–14 of each 21-day cycle. Those in Cohort C received Len 10 mg on D1–14, beginning in C4. Patients in Cohort E1 received ascending doses of Len, starting with 15 mg and either escalated to 25 mg or reduced to 10 mg if not tolerated, on D1–21 of each 28-day cycle. Patients in Cohort F1 received pomalidomide (Pom) 2 or 4 mg PO on D1–21 of each 28-day cycle. Patients were randomized between Cohorts F2 and F3; those in F2 received Atezo as described above and Pom at the maximum tolerated dose (MTD) as determined in Cohort F1. Patients in Cohort F3 did not receive Atezo but were given Pom at the MTD determined in Cohort F1 and dexamethasone (Dex) 20 or 40 mg PO every 7 days from D1 of each cycle. All treatments were continued until loss of clinical benefit, withdrawal, or study end.

## Biomarker assessments

**CyTOF staining and acquisition**: Samples were thawed in a 37⁰C water bath and immediately transferred into RPMI medium +10% fetal bovine serum containing benzonase at 25 U/ml. Cells were pelleted by centrifugation at 300 g for 5 minutes and all supernatant was removed. Longitudinal timepoint samples from the same patient were then barcoded using beta-2 microglobulin antibody conjugated to unique cadmium isotopes and pooled. The pooled patient samples were then stained with a cocktail of surface antibodies (supplemental Table 2) including Cell-ID Rh103 Intercalator (Fluidigm, CA, USA) to label non-viable cells as well as FC receptor blocking solution (BioLegend, London, UK) for 30 minutes at room temperature. All antibodies were either conjugated in-house using Fluidigm's ×8 polymer conjugation kits or purchased commercially from Fluidigm. To further evaluate cell death during staining, a second visibility stain was performed using a 2.5 µM solution of Cell-ID Cisplatin (Fluidigm) in phosphate buffered saline for 2 minutes at room temperature. Next, samples were fixed and barcoded using Fluidigm’s 20-Plex Pd barcoding kit and pooled into a single tube. The pooled sample was then fixed and permeabilized using the Foxp3 Transcription Factor Staining Buffer Set (eBioscience, CA, USA), blocked with heparin at a concentration of 100 U/ml to prevent non-specific staining of eosinophils and stained with a cocktail of intracellular antibodies. Finally, the sample was re-fixed with freshly diluted 2.4% formaldehyde in PBS containing 0.02% saponin and Cell-ID Intercalator-Ir (Fluidigm) to label nucleated cells. The sample was then stored as a pellet in PBS until acquisition. Immediately prior to acquisition, the pooled sample was washed with Cell Staining Buffer and Cell Acquisition Solution (Fluidigm) and resuspended in Cell Acquisition Solution at a concentration of 1 million cells per ml containing a 1:20 dilution of EQ normalization beads (Fluidigm). The sample was acquired on the Fluidigm Helios mass cytometer using the wide bore injector configuration at an acquisition speed of <400 cells per second. The resulting FCS files were normalized and concatenated using Fluidigm's CyTOF software and then de-multiplexed using Astrolabe Diagnostics, Inc., a commercial, cloud-based platform for single-cell analysis.

**Proteomics**: serum profiling using proximity extension multiplex assay. Serum samples were analyzed for a panel of circulating proteins using Olink multiplex assay (Olink Bioscience, Uppsala, Sweden) according to the manufacturer’s instructions. The Immuno-Oncology Panel included 92 proteins associated with human inflammatory conditions. Briefly, an incubation master mix containing pairs of oligonucleotide-labeled antibodies to each protein, was added to the samples and incubated for 16 hours at 4°C. Each protein was targeted with two different epitope-specific antibodies increasing the specificity of the assay. Presence of the target protein in the sample would bring the partner probes in close proximity, allowing the formation of a double strand oligonucleotide polymerase chain reaction (PCR) target. The next day, the extension master mix in the sample initiated the specific target sequences to be detected and generate amplicons using PCR in a 96 well plate. For the detection of the specific protein, a dynamic array integrated fluidic circuit (IFC) 96x96 chip was primed, loaded with 92 protein specific primers and mixed with sample amplicons including 3 inter-plate controls and 3 negative controls. Real-time microfluidic quantitative PCR is performed using the Biomark system (Fluidigm) for the target protein quantification. Data is analyzed using real-time PCR analysis software via the ΔΔCt method and Normalized Protein Expression (NPX) manager. Data were normalized using internal controls in every single sample, inter-plate controls, negative controls and a correction factor and expressed as Log2 scale, which was proportional to the protein concentration. One NPX difference equals the doubling of the protein concentration.

# Results

## Adverse events (AEs) leading to withdrawal from treatment

No AEs that led to withdrawal of study treatment were reported for Cohorts A, B1 and C. AEs led to withdrawal of treatment for 2 patients in Cohort D2 (dysphagia and pneumonia), 1 patient in Cohort D3 (anemia), 1 patient in Cohort E1 (thrombocytopenia), 2 patients in Cohort F2 (neutrophil count decreased and vitiligo) and 1 patient in Cohort F3 (pneumonia).

# Supplementary Tables

## Supplemental Table 1. Summary of patient cohorts

**Supplemental Table 1. Summary of patient cohorts**

| **Cohort** | **Treatment** | **Description** | **Key inclusion criteria** |
| --- | --- | --- | --- |
| **A** | Atezo monotherapy |  | - Must have received 1–3 prior lines of therapy |
| **B1** | Atezo+Len | Dose escalation | - Must have received 1–3 prior lines of therapy |
| **C** | Atezo+Len | Post-ASCT | - Must have received 1–3 prior lines of therapy - Sufficient recovery from 1^st^ or 2^nd^ ASCT within 60–120 days of transplant |
| **D1** | Atezo+Dara | Dara-naïve, run-in | - No prior treatment with an anti-CD38 therapy - Must have received 1–3 prior lines of therapy |
| **D2** | Atezo+Dara | Dara-naïve, dose expansion | - No prior treatment with an anti-CD38 therapy - Must have received 2–3 prior lines of therapy - Refractory to last line |
| **D3** | Atezo+Dara | Dara-refractory | - Must have received ≥2 prior line of treatment - Refractory to a PI and an IMiD - Refractory to Dara |
| **E1** | Atezo+Dara+Len | Dara-naïve | - No prior treatment with an anti-CD38 therapy - Must have received 1–3 prior lines of therapy |
| **F1** | Atezo+Dara+Pom | Dose escalation | - Must have received ≥4 prior lines of treatment - Refractory to last line - No prior treatment with an anti-CD38 therapy |
| **F2** | Atezo+Dara+Pom | Dose expansion | - Must have received ≥4 prior lines of treatment - Refractory to last line - No prior treatment with an anti-CD38 therapy |
| **F3** | Dara+Pom+Dex | Control | - Must have received ≥4 prior lines of treatment - Refractory to last line - No prior treatment with an anti-CD38 therapy |

ASCT, autologous stem cell transplant; Atezo, atezolizumab; Dara, daratumumab; Dex, dexamethasone; IMiD, immunomodulatory drug; Len, lenalidomide; PI, proteasome inhibitor; Pom, pomalidomide.

## Supplemental Table 2. CyTOF antibody panel

| **Metal** | **Marker** | **Clone** | **Manufacturer** | **Catalogue** |
| --- | --- | --- | --- | --- |
| 89 Y | CD45 | HI30 | Fluidigm | 3089003B |
| 113 In | CD57 | HCD57 | BioLegend | 322302 |
| 115 In | CD11c | Bu15 | BioLegend | 337202 |
| 141 Pr | CD103 | Ber-Act8 | BioLegend | 350202 |
| 142 Nd | CD19 | REA675 | Miltenyi Biotec | 130-122-301 |
| 143 Nd | CD45RA | REA562 | Miltenyi Biotec | 130-122-292 |
| 144 Nd | KLRG1 | SA231A2 | BioLegend | 367702 |
| 145 Nd | CD4 | REA623 | Miltenyi Biotec | 130-122-283 |
| 146 Nd | CD8 | REA734 | Miltenyi Biotec | 130-122-281 |
| 147 Sm | ICOS | C398.4A | BioLegend | 313512 |
| 148 Nd | CD16 | REA423 | Miltenyi Biotec | 130-108-0271 |
| 149 Sm | CD127 | A019D5 | Fluidigm | 3149011B |
| 150 Nd | CD1c | REA694 | Miltenyi Biotec | 130-122-298 |
| 151 Eu | CD123 | REA918 | Miltenyi Biotec | 130-122-297 |
| 152 Sm | CD66b | REA306 | Miltenyi Biotec | 130-108-019 |
| 153 Eu | TIGIT | MBSA43 | Fluidigm | 3153019B |
| 154 Sm | TIM3 (CD366) | F38-2E2 | Fluidigm | 3154010B |
| 155 Gd | CD27 | REA499 | Miltenyi Biotec | 130-122-295 |
| 156 Gd | PD-L1 | 29E.2A3 | BioLegend | 329710 |
| 158 Gd | CD33 | WM53 | BioLegend | 303402 |
| 159 Tb | CD138 | 115 | BioLegend | 356502 |
| 160 Gd | CD14 | REA599 | Miltenyi Biotec | 130-122-290 |
| 161 Dy | CD56 | REA196 | Miltenyi Biotec | 130-108-016 |
| 162 Dy | NKG2A | REA797 | Miltenyi Biotec | 120-014-229 |
| 163 Dy | CD5 | UCHT2 | BioLegend | 300602 |
| 164 Dy | FcHR5 | Polyclonal | R&D | AF2087 |
| 165 Ho | NKG2D | REA797 | Miltenyi Biotec | 120-014-229 |
| 166 Er | CD25 | M-A251 | BioLegend | 356102 |
| 167 Er | CCR7 | G043H7 | BioLegend | 353256 |
| 168 Er | CD3 | REA613 | Miltenyi Biotec | 130-122-282 |
| 169 Tm | Tbet | 4B10 | BioLegend | 644802 |
| 170 Er | CD38 | REA671 | Miltenyi Biotec | 130-122-288 |
| 171 Yb | CD39 | A1 | BioLegend | 328202 |
| 172 Yb | CD28 | CD28.2 | BioLegend | 302902 |
| 173 Yb | DNAM1 | 11A8 | BioLegend | 338302 |
| 174 Yb | HLADR | REA805 | Miltenyi Biotec | 130-122-299 |
| 175 Lu | PD-1 | EH12.2H7 | Fluidigm | 3175008B |
| 176 Yb | Granzyme | REA226 | Miltenyi Biotec | 130-108-055 |
| 209 Bi | CD11b | ICRF44 | Fluidigm | 3209003B |

## Supplemental Table 3. Adverse event summary

| **N (%)** | **All patients (n=79)*** |
| --- | --- |
| **Any-grade AEs (in >10% of patients)** |  |
| **Total number of patients with at least one AE** | 79 (100) |
| **Fatigue** | 30 (38) |
| **Diarrhea** | 27 (34) |
| **Neutropenia** | 26 (33) |
| **Thrombocytopenia** | 24 (30) |
| **Anemia** | 22 (28) |
| **Arthralgia** | 20 (25) |
| **Cough** | 20 (25) |
| **Leukopenia** | 20 (25) |
| **Infusion-related reaction** | 19 (24) |
| **Lymphopenia** | 17 (22) |
| **Nausea** | 16 (20) |
| **Upper respiratory tract infection** | 16 (20) |
| **Dyspnoea** | 15 (19) |
| **Headache** | 15 (19) |
| **Pain in extremity** | 14 (18) |
| **Pyrexia** | 14 (18) |
| **Vomiting** | 12 (15) |
| **Constipation** | 11 (14) |
| **Pneumonia** | 11 (14) |
| **Muscle spasms** | 10 (13) |
| **Back pain** | 9 (11) |
| **Insomnia** | 9 (11) |
| **Rash** | 9 (11) |
| **Alanine aminotransferase increased** | 8 (10) |
| **Aspartate aminotransferase increased** | 8 (10) |
| **Chest pain** | 8 (10) |
| **Chills** | 8 (10) |
| **Decreased appetite** | 8 (10) |
| **Grade 3–5 AEs (in >5% of patients)** |  |
| **Total number of patients with at least one AE** | 47 (60) |
| **Neutropenia** | 15 (19) |
| **Pneumonia** | 9 (11) |
| **Anemia** | 7 (9) |
| **Lymphopenia** | 7 (9) |
| **Thrombocytopenia** | 6 (8) |
| **Pain in extremity** | 5 (6) |
| **Arthralgia** | 4 (5) |
| **Febrile neutropenia** | 4 (5) |
| **Hypertension** | 4 (5) |

*Not including Cohort F3 (n=6; patients did not receive Atezo)

AE, adverse event

## Supplemental Table 4. Median treatment duration and number of doses for each agent

|  | **Cohort*** | | | | | | | | | |
| --- | --- | --- | --- | --- | --- | --- | --- | --- | --- | --- |
|  | **A (n=6)** | **B1**  **(n=9)** | **C**  **(n=9)** | **D1**  **(n=6)** | **D2**  **(n=15)** | **D3**  **(n=15)** | **E1**  **(n=7)** | **F1**  **(n=6)** | **F2**  **(n=6)** | **F3**  **(n=6)** |
| **Atezo** |  |  |  |  |  |  |  |  |  |  |
| Median treatment duration, months (range) | 3.81  (2.1–6.9) | 3.55  (2.4–25.2) | 2.10  (1.4–16.4 | 41.51  (8.8–53.8) | 3.19  (0.0–11.0) | 1.38  (0.0–12.4) | 5.52  (0.0–42.8) | 22.62  (3.4–42.1) | 11.99  (1.8–31.3) |  |
| Median number of doses (range) | 6.5  (4–11) | 6.0  (4–37) | 4.0  (3–23) | 81.0 (19–118) | 8.0 (1–23) | 4.0 (1–27) | 13.0 (1–91) | 22.62 (3.4–42.1) | 11.99 (1.8–31.3) |  |
| **Dara** |  |  |  |  |  |  |  |  |  |  |
| Median treatment duration, months (range) | - | - | - | 41.30 (8.3–53.4) | 3.22  (0.0–11.1) | 1.68  (0.0–12.0) | 5.55 (0.3–42.3) | 22.19  (3.5–41.9) | 11.79  (1.8–31.4) | 2.79  (1.0–21.7) |
| Median number of doses (range) | - | - | - | 52.5  (20–69) | 12.0 (1–21) | 8.0 (1–24) | 16.0 (2–57) | 35.0  (10–56) | 23.5  (8–42) | 9.5  (4–33) |
| **Len** |  |  |  |  |  |  |  |  |  |  |
| Median treatment duration, months (range) | - | 4.17 (4.0–25.6) | 3.98 (2.6–25.6) | - |  | - | 5.82 (0.4–42.8) | - | - | - |
| Median number of doses (range) | - | 84.0 (83–516) | 83.0 (48–516) | - | - | - | 131.0 (1–951) | - | - | - |
| **Pom** |  |  |  |  |  |  |  |  |  |  |
| Median treatment duration, months (range) | - | - | - | - | - | - | - | 22.85 (3.6–42.1) | 10.50 (2.3–31.6) | 2.64 (1.0–22.4) |
| Median number of doses (range) | - | - | - | - | - | - | - | 524.0 (78–955) | 196.5 (57–661) | 59.5 (13–499) |

Atezo, atezolizumab; Dara, daratumumab; Dex, dexamethasone; Len, lenalidomide; Pom, pomalidomide.

*Cohort A: Atezo monotherapy; B1: Atezo+Len; C: Atezo+Len; D1/D2/D3: Atezo+Dara; E1: Atezo+Dara+Len; F1/F2: Atezo+Dara+Pom; F3: Dara+Pom+D

## Supplemental Table 5. CyTOF analysis to identify cellular phenotypes associated with cluster D3 (patients with Dara-refractory disease).

See separate Excel file

## Supplemental Table 6. Pathway Enrichment Analysis using the Panther Overrepresentation Test (Pantherdb.org) for significant genes (t-test p-value<0.01) that had higher expression in CD138nRNA3.1 (enrichment in CD138nRNA3.1) or other clusters (enrichment in CD138nRNA3.2 and 3.3).

See separate Excel file

# Supplementary Figures

## Supplemental Figure 1. Schematic of the SNF algorithm. Step 1: Patient similarity matrices are constructed for each data type using pairwise correlation. Step 2: Patient similarity matrices are equivalent to patient similarity networks, whereby the nodes represent patients and lines represent patients’ pairwise similarities. Step 3: Each patient similarity network is updated iteratively with the information from the other networks, making them more similar with each step. Step 4: Represents the final fused network of patients to which the SNF process has converged. SNF, similarity fusion network.


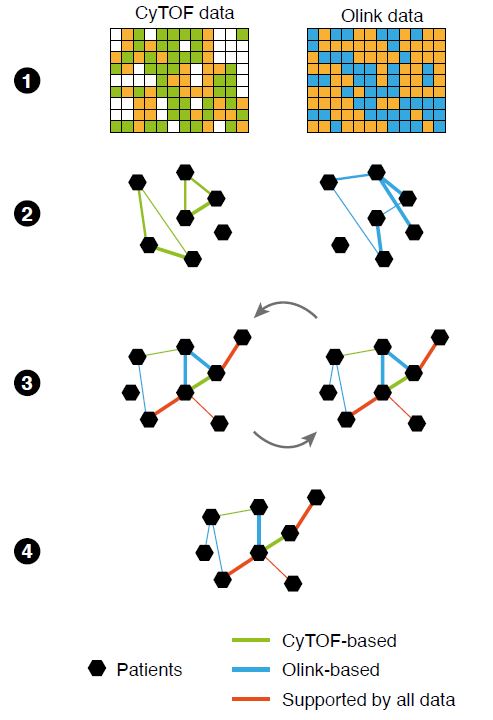


## Supplementary Figure 2. Pharmacodynamic response to Atezo monotherapy. Atezo, atezolizumab; C, cycle; D, day; EOT, end of treatment.

**
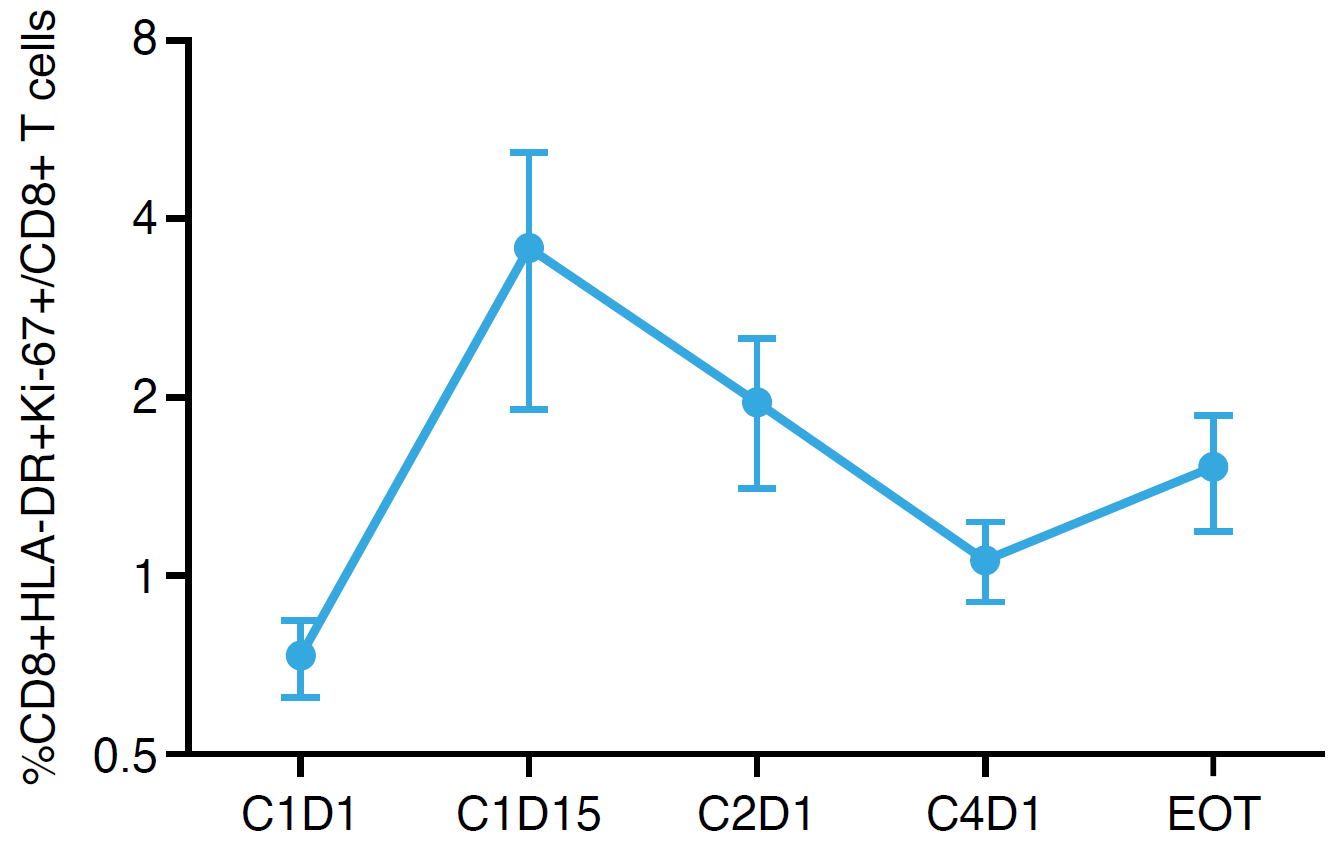
**

## Supplemental Figure 3. Baseline and post-treatment T-cell activation and proliferation observed in Dara-naïve and Dara-refractory responders and non-responders treated with Atezo+Dara in A) PB, and B) BMA

### A


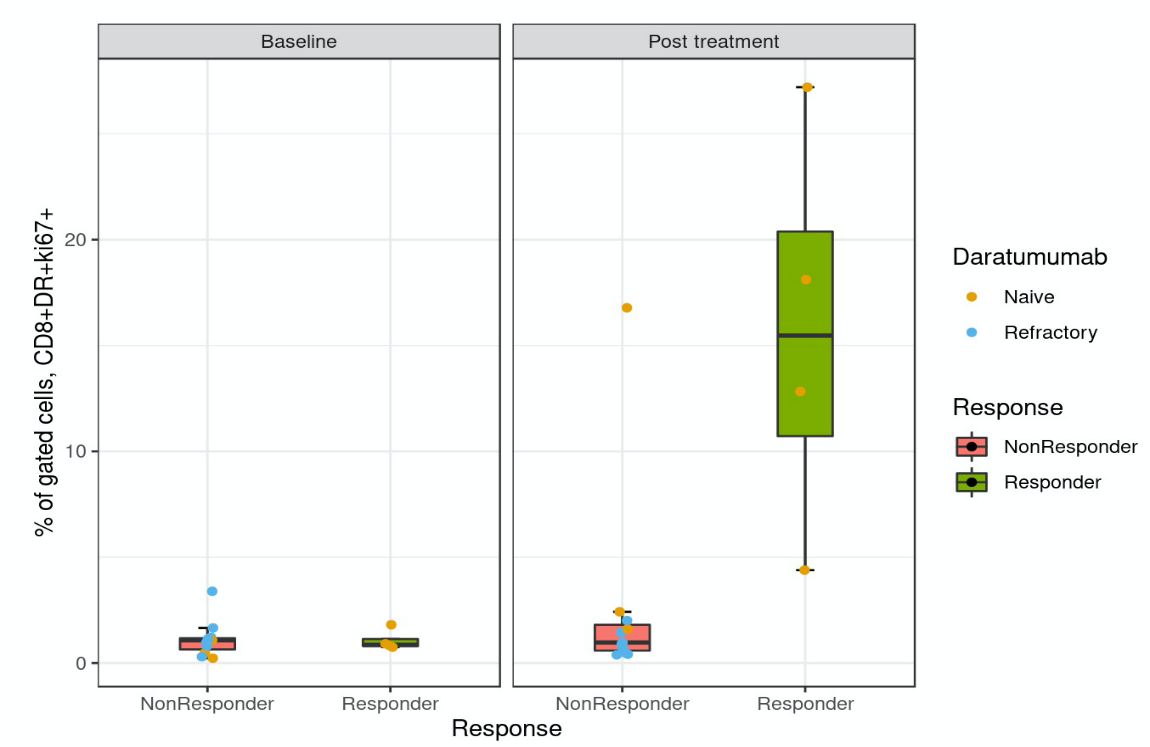


### B


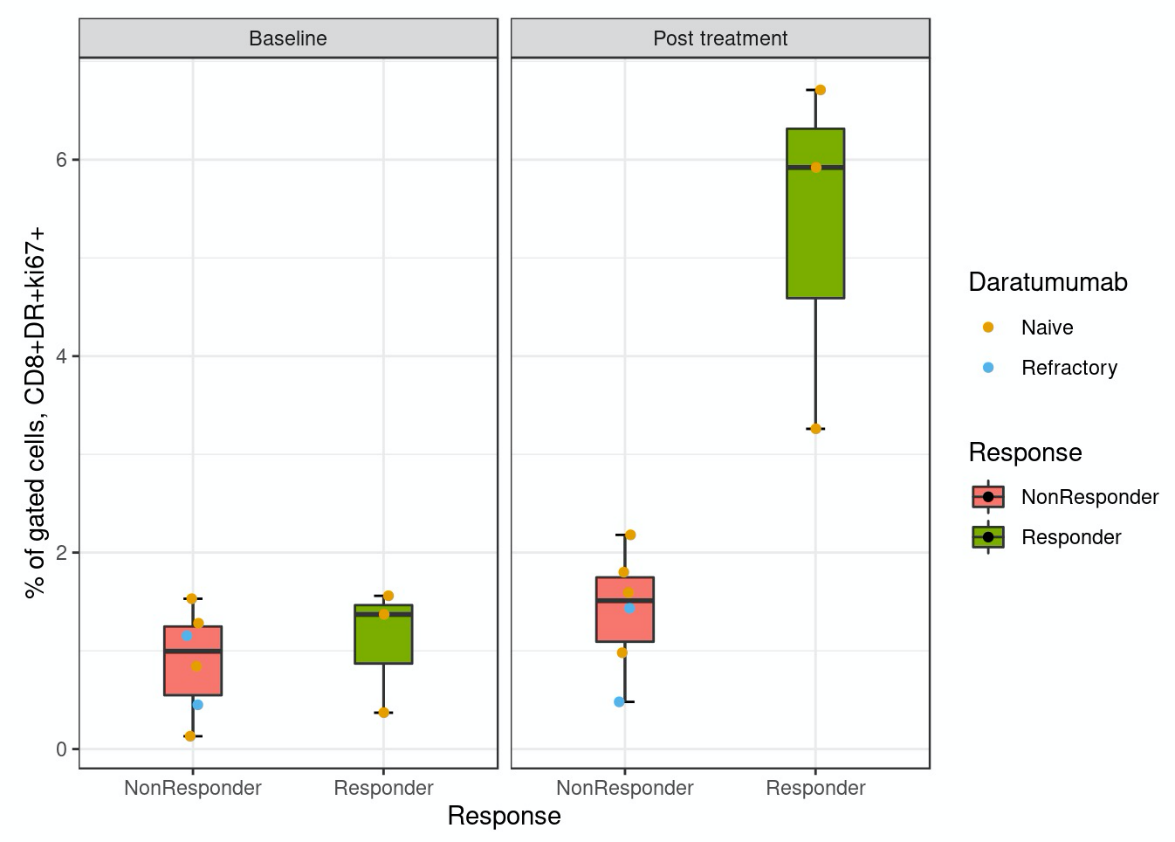


Atezo, atezolizumab; BMA, bone marrow aspirates; Dara, daratumumab.

## Supplemental Figure 4. Baseline osteoclast density in A) non-responders vs responders, and B) Dara-naïve vs Dara-refractory patients treated with Atezo+Dara

### A

**
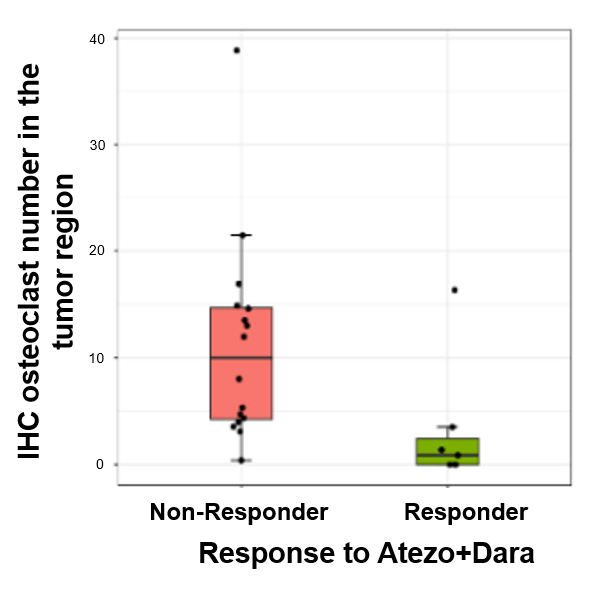
**

### B

**
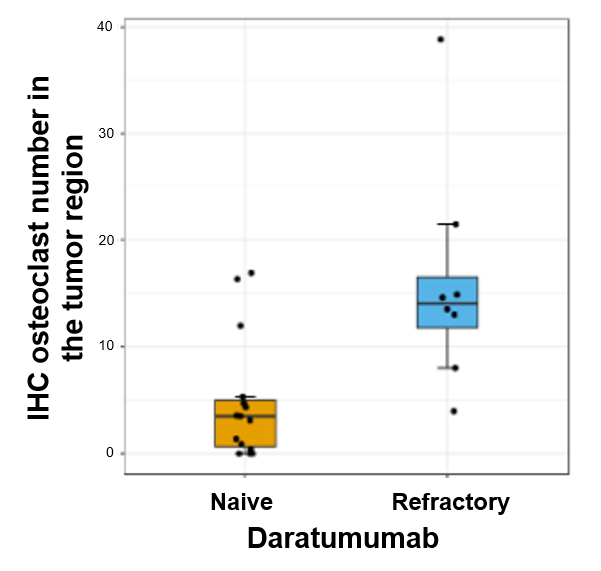
**

Atezo, atezolizumab; Dara, daratumumab; IHC, immunohistochemistry.

## Supplemental Figure 5. Group association with overall survival (OS) and duration of response (DOR) A) CyTOF derived groups association with OS. B) SNF derived groups association with OS. C) CyTOF derived groups association with DOR D) SNF derived groups association with DOR

### A


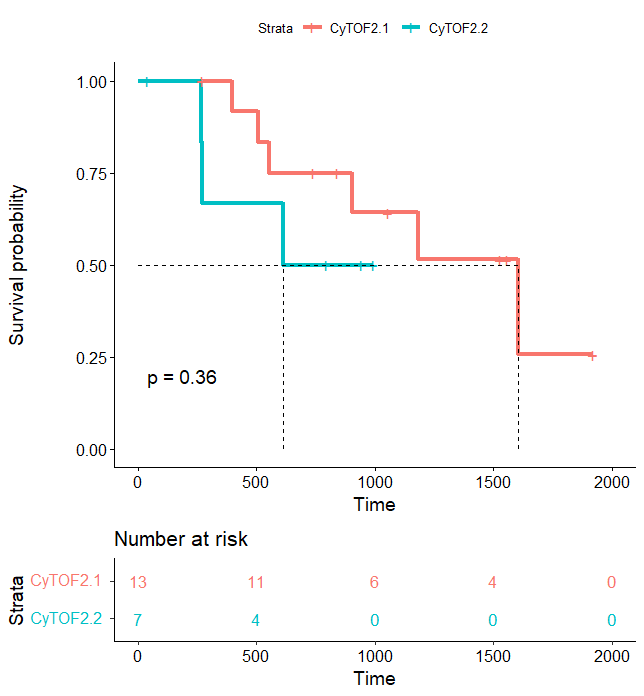


### B


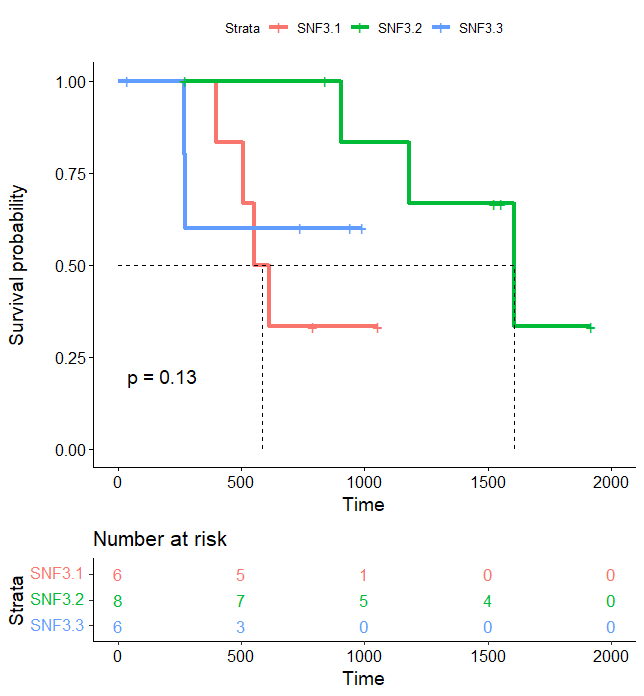


### C


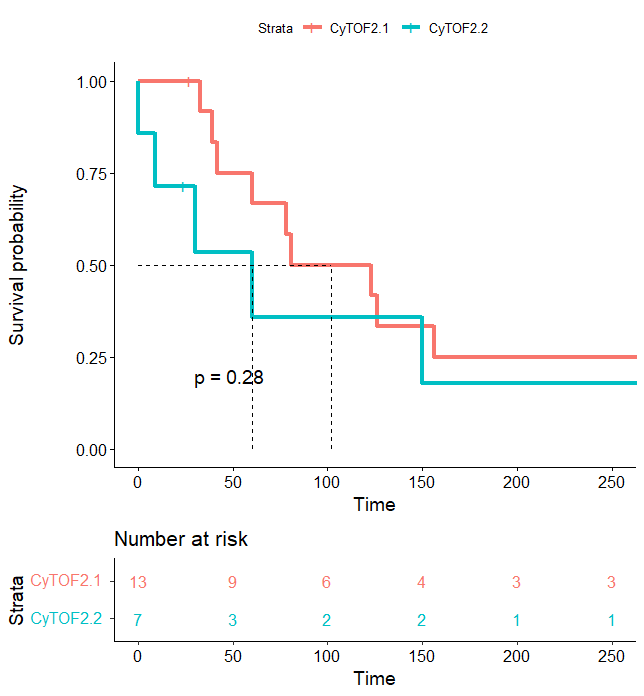


### D


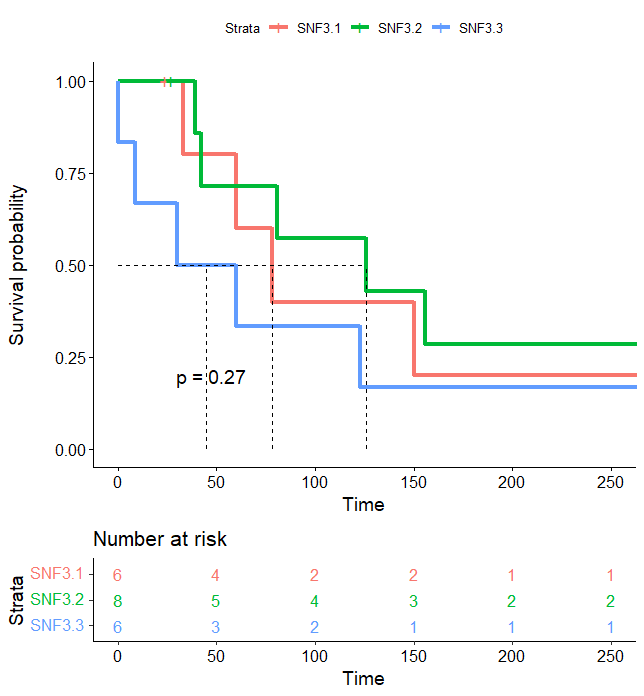

Supplement: Supplementary file 1 [file DataSheet_1.docx]
